# Immunohistochemical and molecular analyses of HER2 status in breast cancers are highly concordant and complementary approaches

**DOI:** 10.1038/bjc.2011.135

**Published:** 2011-05-03

**Authors:** J Lehmann-Che, F Amira-Bouhidel, E Turpin, M Antoine, H Soliman, L Legres, C Bocquet, R Bernoud, E Flandre, M Varna, A de Roquancourt, L-F Plassa, S Giacchetti, M Espié, C de Bazelaire, L Cahen-Doidy, E Bourstyn, A Janin, H de Thé, P Bertheau

**Affiliations:** 1 AP-HP, Hosp Saint-Louis, Department of Biochemistry, Paris 75010, France; 2 CNRS UMR7212/INSERMU944, Paris 75010, France; 3 Univ Paris Diderot, Sorbonne Paris Cité, Paris 75010, France; 4 AP-HP, Hosp Saint-Louis, Department of Pathology, Paris 75010, France; 5 INSERM UMR_S728, Paris 75010, France; 6 AP-HP, Hosp Tenon, Department of Pathology, Paris 75010, France; 7 AP-HP, Hosp Saint-Louis, Breast Diseases Center, Paris 75010, France; 8 AP-HP, Hosp Saint-Louis, Department of Radiology, Paris 75010, France; 9 AP-HP, Hosp Saint-Louis, Department of Surgery, Paris 75010, France

**Keywords:** HER2, HER2/neu, ERBB2, c-erb-b2, heterogeneity, real-time PCR

## Abstract

**Background:**

Immunohistochemistry (IHC) and fluorescent *in situ* hybridisation (FISH) are currently the most commonly used methods to assess HER2 status. PCR-based assays allow quantitative determination of HER2 amplification (Q-PCR) or overexpression (Q-RT–PCR), but are not routinely used. We evaluated the relevance of Q-RT–PCR for HER2 status determination.

**Methods:**

We compared IHC and Q-RT–PCR in 466 breast tumours. In discordant or equivocal cases, five additional methods (IHC with two other antibodies, FISH, silver *in situ* hybridisation (SISH) and Q-PCR) were combined to determine HER2 status. Two cases with HER2 intra-tumour heterogeneity were further explored by allelic profiles analysis and HUMARA clonality determination after microdissection.

**Results:**

We observed 97.3% concordance between Q-RT–PCR and non-equivocal IHC. Twelve out of 466 cases (3%) revealed discordances between the two methods. The power of Q-RT–PCR to predict HER2 status (defined by seven methods) was similar to that of IHC. Although rare, some discordances between techniques might be due to HER2 intra-tumour heterogeneity and we report two examples, one tumour containing two distinct clones, another tumour consisting of HER2 amplified and non-amplified subclones.

**Conclusion:**

Q-RT–PCR and IHC are highly concordant methods for HER2 status assessment, and Q-RT–PCR allows a highly reliable quantitative assessment and could be a useful adjunct to IHC.

The HER2 gene encodes the human epidermal growth factor receptor 2 with tyrosine kinase activity (Slamon *et al*, 1987) and is overexpressed in 15–20% of breast cancers (Owens *et al*, 2004), through gene amplification with well correlated level of protein expression (Pauletti *et al*, 1996; Yarden, 2001). This molecular abnormality defines breast tumours with poor prognosis and increased risk of early relapse (Slamon *et al*, 1987, 1989), but predicts response to the humanised monoclonal anti-HER2 antibody trastuzumab (Herceptin) or to small tyrosine kinase inhibitors such as lapatinib or erlotinib.

The increasing number of patients with breast cancer whose survival has been improved by trastuzumab treatment underlines the need for sensitive, specific, highly reproducible and cost-efficient methods to identify patients eligible for anti-HER2 therapies. Furthermore, HER2 status not only predicts anti-HER2 efficacy, but could also determine other treatment options. Indeed, HER2-overexpressing breast tumours are often resistant to hormonotherapy and more sensitive to anthracycline-based and taxane-containing chemotherapy (Paik *et al*, 2000; Pritchard *et al*, 2008), so that all invasive breast cancer need an HER2 evaluation at diagnosis.

In daily practice, according to the American Society of Clinical Oncology/College of American Pathologists (ASCO/CAPs) recommandations (Wolff *et al*, 2007), HER2 status is determined by immunohistochemistry (IHC) followed, if necessary, by fluorescent *in situ* hybridisation (FISH), although FISH first-line determination is also encouraged by some authors (Sauter *et al*, 2009). Immunohistochemistry results are obtained on formalin-fixed paraffin-embedded (FFPE) samples and expressed as a four-scale score system (0 to 3+). Determination of HER2 status by FISH is also performed on FFPE samples. It shows the mean number of HER2 copies using a DNA probe hybridising to the HER2 gene alone or in association with a centromeric probe as control for chromosome 17 copy number expressed as HER2/CEN17 ratio. Fluorescent *in situ* hybridisation is now challenged by chromogenic (CISH) or silver *in situ* hybridisation (SISH), faster methods using a chromogenic signal that do not decay over time, that can be further reevaluated and need only a classical light microscope (Isola *et al*, 2004; Laakso *et al*, 2006; Papouchado *et al*, 2010). A ‘gold standard’ for HER2 determination does not really exist and the ASCO/CAPs study estimates that 20% of current HER2 tests may be inaccurate owing to multiple preanalytic and analytic variables (Wolff *et al*, 2007). Indeed, the existence of various IHC protocols, FDA-approved antibodies or probes contributes to interlaboratory variability. Improvement in HER2-testing reproducibility between laboratories is crucial and new HER2-testing technologies are also needed.

Different studies have evaluated the performance of Q-RT–PCR or Q-PCR to determine the HER2 status (Laudadio *et al*, 2007). Quantitative PCR using primers flanking the HER2 gene or mRNA can quantify gene amplification or messenger overexpression. PCR-based assays are easy, rapid, sensitive, specific and quantitative approaches without the inherent inter-observer variability of IHC and FISH/CISH techniques. It can be used on small samples and can be standardised and automated. DNA extraction can also be performed on FFPE tissues. However, RT–PCR technology, more sensitive to RNA quality, is more robust if frozen tissues are used. The main PCR drawback is that DNA or RNA extraction mixes tumour and non-tumour cells and can lead to tumour cells dilution with risk of false negative. Conversely, the mix of invasive tumour cells with high-grade intraductal HER2-positive tumour cells can lead to false positive results. Provided that sample purity is controlled with microscopical examination, PCR-based determination has been shown to correlate well with IHC and FISH (Vinatzer *et al*, 2005; Barberis *et al*, 2008).

In order to evaluate the benefits of PCR-based technology in daily HER2 testing, we performed a large prospective study comparing HER2 determination with IHC and Q-RT–PCR. As recommended in guidelines, IHC 2+ cases were further explored not only by FISH and SISH but also by other IHC tests and Q-PCR. Moreover, all discordant cases were explored by these additional techniques. We also analysed extensively two cases showing striking HER2 intra-tumour heterogenity that may explain some technical discordances.

We observed that molecular approaches are powerful and reliable quantitative tools for HER2 status assessment that could complement IHC for optimal patient care.

## Materials and methods

### Patients and samples

We analysed 466 primary breast tumours obtained from patients treated in Saint Louis Hospital (Paris) from 2002 to 2007. All patients were informed of the study according to our Institutional Review Board recommendations. A total of 332 samples were obtained from surgical specimen and 134 were obtained with fine-needle biopsies. Tumours with >10% *in situ* component were excluded from this study. Haematoxylin–eosin (H&E) stainings, immunohistochemical stainings and *in situ* hybridisation techniques were performed on FFPE tissue samples. Q-RT–PCR and Q-PCR were performed on RNA and DNA extracted from frozen tissues.

### IHC detection

HER2 immunohistochemistry was performed with the monoclonal HER2 CB11 antibody (Novocastra, Newcastle upon Tyne, UK, dilution 1/250) in the BenchmarkXT immunostainer (Roche Diagnostics, Basel, Switzerland) with calibrated positive controls and internal (on slide) negative controls. Evaluation of immunostainings was performed by two pathologists (PB, AR) and scored according to ASCO guidelines (Wolff *et al*, 2007; Gown, 2008): negative for 0 (no membrane staining) and 1+ (faint or barely perceptible incomplete membrane staining); equivocal for 2+ (10–30% tumour cells with strong complete membrane staining or >10% tumour cells with moderate complete membrane staining) and positive for 3+ (>30% tumour cells with strong complete membrane staining).

For discordant and CB11 equivocal cases, HER2 immunohistochemistry was performed with A0485 polyclonal (Dako, Glostrup, Denmark, dilution 1/500) and 4B5 monoclonal antibody (Roche Diagnostics, prediluted) using the Discovery immunostainer (Roche Diagnostics). HER2 scores were evaluated as described below.

Other antibodies were used with the Discovery immunostainer: oestrogen receptor (Novocastra, clone 6F11, dilution 1/50), progesteron receptor (Novocastra, clone 312, dilution 1/75), cytokeratin 5/6 (Dako, clone B4, dilution 1/50), cytokeratin 17 (Dako, clone E30, dilution 1/50) and cytokeratin 8 (Millipore, Billerica, MA, USA, clone MAB 3414, 1/50).

### SISH and FISH detection

Silver *in situ* hybridisation and FISH were performed on 3 *μ*m paraffin tissue sections. Silver *in situ* hybridisation staining, with HER2 and chromosome 17 probes, was performed in BenchmarkXT slide stainers (Roche Diagnostics) and described in Dietel *et al* (2007). Fluorescent *in situ* hybridisation staining was performed using the Zytolight Spec HER2/CEN17 kit (Zytovision, CliniScience, Montrouge, France) according to the manufacturer's protocol. Fluorescence signal were counted by one pathologist (MA) using a (Leica DM 4000) Zeiss Axioscope Imager Z1 fluorescence microscope (Zeiss, Oberkochen, Germany). A minimum of 80 tumour cell nuclei, with intact morphology according to DAPI counterstaining, were counted. The HER2/CEN17 ratio was obtained by dividing the mean number of HER2 signals by the mean number of CEN17 signals in tumour cells and defined HER2 gene amplification if >2.2, equivocal if between 1.8 and 2.2 and no HER2 amplification if <1.8, according to ASCO/CAPs recommendations (Wolff *et al*, 2007).

### Quantification of HER2 overexpression by Q-RT–PCR and HER2 gene copy number by Q-PCR

Nucleic acids were extracted by phenol/chloroform procedure. Tumour cell purity and presence of *in situ* carcinoma were assessed on adjacent H&E-stained sections. Quantitative PCR were performed on LightCycler 2.1 instrument (Roche Diagnostics). HER2 overexpression was evaluated by relative quantification using TATA-binding protein as endogen control (Bossard *et al*, 2005). Final result was expressed as a normalised ratio considered as over-expressed if >7. The cut-off ratio was determined on a tumours training set using univariate partition method (XLSTAT software) and correlation with IHC–HER2 expression. HER2 amplification was evaluated on DNA using the LightCycler-HER-2/neu DNA Quantification kit (Roche Diagnostics) in all IHC2+ and IHC/Q-RT–PCR discordant specimens. The assay amplifies simultaneously one HER2 fragment and one gastrin fragment, the reference gene localised on the chromosome 17. Results were expressed as the ratio of HER2/gastrin in the sample, normalised with the same ratio in the calibrator DNA set. A ratio above 2 was considered amplified. Results between 2 and 3 were repeated.

### Allelic profiles analysis

Allelic profiles were analysed as described in Varna *et al* (2007). We used a PALM Microbeam/Olympus system to perform laser tissue microdissection on FFPE tissue sections. PCR was performed directly on cell lysates with at least 500 cells for each PCR. Five microsatellite dinucleotide repeats were used: D17S250, D17S855, D17S1840, D13S153 and D9S171. Whole tumour allelic profiles and microdissected areas allelic profiles were compared.

### Clonality assessment using androgen receptor gene methylation pattern

The androgen receptor gene (HUMARA) polymorphism is characterised by highly polymorphic short-tandem CAG repeat units, 100 bp downstream of a methylated site in the coding region of its first exon (Lucas *et al*, 1997; Fujita *et al*, 1998; Wang *et al*, 2007). Before digesting the genomic DNA with methylation-sensitive restriction enzyme HpaII, electrophoresis of heterozygote cases shows two alleles. After digesting the genomic DNA with HpaII, electrophoresis shows two different alleles if the tumour is not monoclonal and only one allele or at least strong allelic imbalance if the tumour is of monoclonal origin due to X-chromosome inactivation mosaicism, as solely the inactive methylated allele is not cut at the restriction site and hence PCR amplified.

Tumour DNA was amplified at the HUMARA locus either with or without HpaII predigestion and overall tumour profiles and microdissected areas profiles were compared.

## Results

### IHC and Q-RT–PCR comparisons

**Figure 1:**
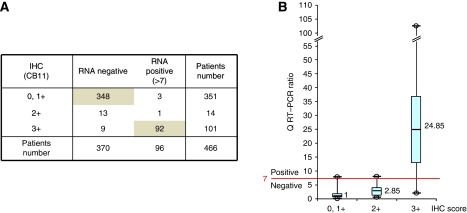
Comparison of HER2 determination by IHC (CB11) and Q-RT–PCR on 466 breast tumours treated in St Louis Hospital. (**A**) Distribution of tumour samples according to HER2 status assessed on formalin-fixed, paraffin-embedded samples with 0/1+, 2+ and 3+ IHC scores and Q-RT–PCR on fresh frozen samples, with a cut-off ratio of 7. (**B**) HER2 Q-RT–PCR ratio according to the three IHC score groups: each box shows the 25–75th percentile (box extremities), the median values (line in the box and value outside) and the lowest and highest values (bottom and top bars of the whisker).

To determine HER2 status, we performed immunohistochemistry with CB11 antibody and Q-RT–PCR on all 466 cases (Figure 1). Overall concordance was excellent (97.3%), especially in IHC 0,1+ subgroup (348 negative Q-RT–PCR/351 IHC 0/1+: 99.2%). Concordance was also good in IHC 3+ cases (91%: 92 positive Q-RT–PCR/101 IHC 3+). However, in 12 out of 466 cases (3%), the two techniques were discordant (either IHC 0,1+/Q-RT–PCR>7 or IHC 3+/Q-RT–PCR<7). Among these 12 discordant cases, 11 had been obtained after surgical procedure and only one after fine-needle biopsy. In the 14 out of 466 IHC 2+ equivocal cases, Q-RT–PCR showed the absence of HER2 overexpression in 13 out of 14 cases (93%).

### Analysis of the 26 discordant or equivocal cases

**Figure 2:**
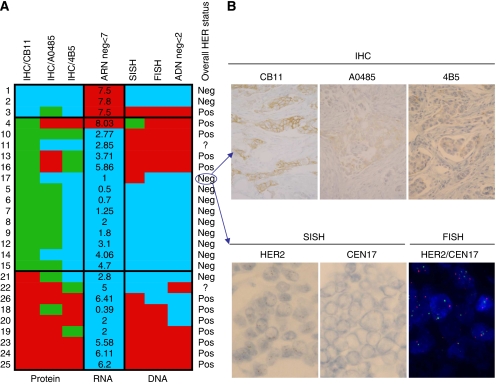
Analysis of 14 equivocal and 12 discordant cases (*n*=26). (**A**) Comparison of seven different methods (columns) in the 26 discordant or equivocal cases (lines): determination at the protein level by IHC – CB11, polyclonal A0485 and 4B5 antibodies, at the mRNA level by Q-RT–PCR, at the DNA level by SISH, FISH and Q-PCR. A negative result is symbolised in blue, positive in red and equivocal in green. Final HER2 status is based on the result of all seven methods: positive if there were majority of positive results, negative if there were majority of negative results, and unclassified in other situations. (**B**) Illustration of discordant case #17: Upper panel: immunohistochemical staining for HER2 with CB11, A0485 and 4B5 antibodies and indirect immunoperoxydase visualisation (magnification × 250). Lower panel: SISH staining with HER2 probe, CEN17 probe and FISH staining with HER2 probe in green and CEN17 probe in orange. (magnification × 400).

According to guidelines, all IHC score 2+ (*n*=14) were analysed not only by hybridisation methods (FISH and SISH), but also with two other IHC–HER2 antibodies and Q-PCR (Figure 2). These additional methods were also performed in all discordant cases (*n*=12). An overall HER2 status was defined for each of these 26 discordant or equivocal cases, based on the results of all seven techniques used, a case being HER2 positive if there were more positive than negative results (12 out of 26 cases), being negative if there were more negative than positive results (12 out of 26 cases) and being HER2 unclassified in other situations (2 out of 26 cases). Among the 14 equivocal cases, 4 were finally scored positive by the overall HER2 status, 9 negative and 1 remained undefined. In these equivocal cases, Q-RT–PCR analysis predicted the final HER2 status in 10 cases and failed in only 3 cases. However, in the 12 discordant cases, Q-RT–PCR predicted final HER2 status in only two cases and failed in nine cases.

The overall ability of Q-RT–PCR to predict final HER2 status in the 24 cases with known final HER2 status was, therefore, 12 out of 24, while the overall ability of IHC CB11 to predict final HER2 status was 9 out of 24. Accordingly, Q-RT–PCR and IHC, respectively, predicted the overall HER2 status in 452 tumours and in 449 tumours. Among the 26 IHC/Q-RT–PCR discordant or IHC 2+ cases, only six tumours (#1, 2, 14, 23, 24 and 25) showed highly concordant results, with 6 out of 7 methods showing similar results. The other 20 cases all showed more extensive discrepancies among the techniques used, with only three to five concordant methods. The results in case #4, either positive or borderline, were possibly due to chromosome 17 polysomy that was demonstrated with FISH and SISH techniques. In case #17 (Figure 2B), we observed heterogeneous IHC staining among the three antibodies used, as well as FISH negativity and moderate amplification with SISH. In the other cases, no easy explanation could be given for these technical discrepancies and either borderline HER2 status or true intra-tumour heterogeneity could be implicated.

### Phenotype and genotype analyses of two cases with HER2 intra-tumour heterogeneity

In order to explore one possible cause of discordances between techniques, we analysed two cases that showed obvious intra-tumour HER2 heterogeneity.

**Figure 3:**
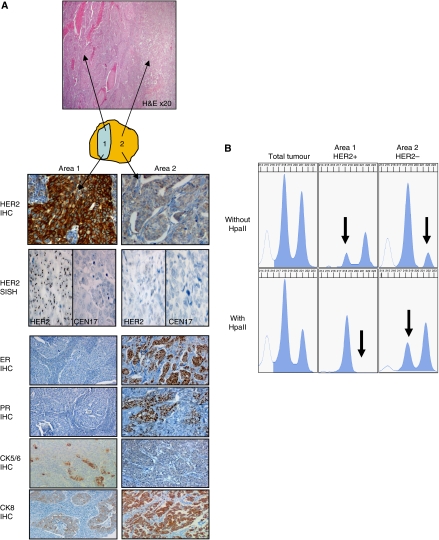
Analysis of case A heterogeneity. (**A**) Schematic representation of case A with distinction of area 1 (HER2+) and area 2 (HER2−): H&E section, immunohistochemical stainings for HER2, ER, PR, CK5/6, CK8 and SISH evaluation of HER2 (magnification × 250). (**B**) Analysis of X-chromosome methylation pattern (HUMARA): allelic profiles for the total tumour and microdissected areas 1 and 2 are shown, before HpaII (upper line) and after HpaII (lower line) digestion. Differences between the two areas are represented by arrows.

In *Case A*, H&E examination showed the presence of a 1-cm less differentiated area (area 1) located inside the main tumour component (area 2) (Figure 3A). Area 1 was scored 3+ for HER2, was positive for CK5/6 and negative for ER, PR, CK8 and CK17, while area 2 was negative for HER2, CK5/6 and CK17 and positive for ER, PR and CK8. Silver *in situ* hybridisation confirmed HER2 amplification only in tumour cells of area 1 (Figure 3A). There was allelic loss at D17S855 in area 1 but not in area 2 (data not shown). Allelic profiles with the other microsatellites were either non-informative or showed no significant difference between the two areas (data not shown). The analysis of the X-chromosome methylation pattern showed important differences between areas 1 and 2 (Figure 3B): before HpaII digestion, allelic profiles showed LOH in each area, but on two distinct alleles. After HpaII digestion, profiles showed inactivation of one X chromosome in both areas 1 and 2, indicating that both areas are populated by monoclonal cells. However, areas 1 and 2 did not inactivate the same X chromosome, demonstrating that they are not deriving from the same clone.

**Figure 4:**
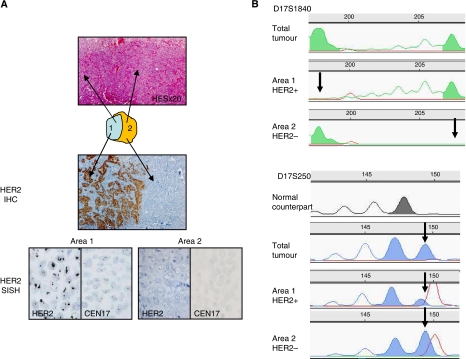
Analysis of case B heterogeneity. (**A**) Schematic representation of case B with distinction of areas 1 and 2: in H&E section, in immunohistochemical stainings for HER2 and SISH evaluation of HER2 (magnification × 250). (**B**) Analysis of allelic profile using D17S1840 and D17S250 microsatellites after microdissection of areas 1 and 2. The profile in grey represents the normal counterpart and the profile in green or blue the different tumour areas and the total tumour before microdissection. The arrows point to loss of heterozygosity (D17S1840) or to new alleles (D17S250).

In *Case B* (case #18 of this study), HER2 immunohistochemical staining (Figure 4A) showed two sharply demarcated areas, one with a strong membranous staining in over 80% of tumour cells (area 1) and one totally negative (area 2). These two areas looked totally similar on H&E staining. Other immunostainings (ER, PR, CK5/6, CK17 and CK8) were similar in both areas (data not shown). Silver *in situ* hybridisation confirmed HER2 amplification in area 1 and lack of amplification in area 2, with sharp borders between the two areas (Figure 4A). Allelic profiles obtained after microdissection with D17S1840 showed only one allele in area 1 and only the other allele in area 2, consistent with subclonal heterogeneity (Figure 4B). For D17S250, one new allele was observed in the non-microdissected tumour, as well as in areas 1 and 2 (Figure 4B), strongly suggesting that areas 1 and 2 are derived from a common tumour cell. Allelic profiles with the three other microsatellites were almost similar in both areas. The analysis of the X-chromosome methylation pattern was not informative since it showed inactivation of the same X chromosome in both areas (data not shown).

## Discussion

Selection of patients for trastuzumab treatment is primarily performed by IHC using HercepTest, CB11 or 4B5 antibody, as recommended (Birner *et al*, 2001; Wolff *et al*, 2007). HER2 immunostaining is easy to perform, available as a standard method in pathology laboratories, widely applicable (on FFPE specimens), very reliable (Lebeau *et al*, 2010; Purdie *et al*, 2010) and relatively inexpensive, but is only a semi-quantitative method. Yet, in 3–15% of cases (Dendukuri *et al*, 2007), IHC is equivocal and further analyses are required, leading to the usual IHC+FISH association. Like IHC, FISH is a semi-quantitative morphological method but with higher costs and need of specialised expertise and equipment. In the context of HER2 status assessment, Q-RT–PCR could also be a useful option and an alternative to the current IHC+FISH procedure. Several studies have already compared IHC and Q-RT–PCR (Cronin *et al*, 2004; Ginestier *et al*, 2004; Gjerdrum *et al*, 2004; Bossard *et al*, 2005; Esteva *et al*, 2005; Vanden Bempt *et al*, 2005; Vinatzer *et al*, 2005; Bergqvist *et al*, 2007; Kostopoulou *et al*, 2007; Barberis *et al*, 2008; Cuadros *et al*, 2010) (Table 1). These reports showed good overall concordance (82–100%) with frozen or FFPE specimen, and mostly without microdissection, but in small patient series. Moreover, HER2 mRNA evaluation was shown to be a fast, reliable and cost-effective alternative to the IHC+FISH procedure and also correlated with overall survival and disease-free survival (Vinatzer *et al*, 2005).

In this prospective study of 466 breast tumours comparing IHC and Q-RT–PCR determination of HER2 status, we show that Q-RT–PCR was very strongly correlated with IHC (overall concordance 97.3%). In the 12 discordant cases, Q-RT–PCR was not as powerful as IHC to predict the final HER2 status determined by five other methods, with seven and one false negative cases and two and one false positive cases for Q-RT–PCR and IHC, respectively. However, four cases (Figure 2A, cases #23, 24, 25 and 26) among the seven Q-RT–PCR false negative cases had ratios very close to the cut-off value, suggesting that tumour cells dilution could explain the discrepancy. Note that in cases #23, 24 and 25, only Q-RT–PCR method was unable to predict the HER2 positivity, perhaps because mRNA analysis can be tricky in borderline cases. Similarly, in the two false positive cases (#1 and #2), the ratio was also close over the cut-off value and #1 presented a small *in situ* component, although evaluated to be <10%.

More importantly, in the 14 IHC equivocal cases, Q-RT–PCR was highly predictive of the final HER2 status in 10 out of 13 cases. These results thus validate the use of Q-RT–PCR as alternative to FISH in IHC 2+ cases. Overall, Q-RT–PCR and IHC had statistically similar efficiency for predicting HER2 status in the overall 466 cases we studied (452 out of 466 and 449 out of 466, respectively).

Q-RT–PCR is quick, easy to perform, quantitative and has no inter-observer variability. However, dilution of tumour genomic material with non-neoplastic tissue or presence of *in situ* component are well-known drawbacks of Q-RT–PCR, so that microscopical control of the sample is particularly crucial prior to molecular extraction. If expert pathological selection is performed, microdissection can clearly be avoided (Gjerdrum *et al*, 2004).

Since Q-RT–PCR can be performed with as little as 100 ng RNA, the corresponding amount of frozen tissue is easily obtained even with 14G or 16G fine-needle biopsies of breast tumours (O'Flynn *et al*, 2010), although a frozen tissue workflow has to be organised. While we determined here the mRNA level using frozen tissues, several reports demonstrated also the good HER2 mRNA/protein concordance in FFPE samples (Cronin *et al*, 2004; Barberis *et al*, 2008).

Immunohistochemistry and Q-RT–PCR are, therefore, two complementary approaches, with an excellent overall sensitivity and with almost no equivocal cases. Measurement of tumour cell percentage and morphological HER2 assessment are done by H&E and IHC stainings, while quantitative HER2 assessment is obtained by Q-RT–PCR, for moderate costs (Vinatzer *et al*, 2005). Indeed, the combination of IHC+Q-RT–PCR has an estimated cost of 157.27 euros, compared with 525.68 euros for IHC+FISH determination (Vinatzer *et al*, 2005). The association of these two techniques in breast cancer would be useful for patient care, each technique controlling and complementing the other one. However, it is not possible to draw similar conclusions in gastroesophageal cancers, now often tested for HER2 status, since these tumours might be more heterogeneous than breast tumours and the use of molecular techniques in this field of pathology should be evaluated.

One rare but significant finding in our study was that a few tumours (12 out of 466) had a highly discordant HER2 status depending on the methods used. One of these discordant cases (tumour #4) showed chromosome 17 polysomy that is well known to complicate HER2 status analysis (Dal Lago *et al*, 2006). Another case (tumour #17) showed highly discordant results among the three antibodies used for IHC, although stainings had been performed on three adjacent tissue sections, maybe reflecting inappropriate fixation of the sample. For the other 10 discordant cases, only hypotheses can be drawn to explain these discrepancies: some of these cases could have a true borderline HER2 status; therefore, being considered negative or positive with only slight technical sensitivity changes.

Intra-tumour heterogeneity may also explain some of these discrepancies, even when the analyses are performed on close tissue areas. We describe here two cases that are typical examples of regional HER2 intra-tumour heterogeneity. In case A, methylation of one X chromosome in HER2+ area and methylation of the other X chromosome in HER2− area strongly suggest the existence of two distinct tumours, deriving from two distinct initiating tumour cells. Case A would be, therefore, like the so-called ‘collision tumours’ described by pathologists (Isaka *et al*, 2007; Kleist *et al*, 2010) all characterised by the simultaneous occurrence at the same place and at the same time of two tumours with distinct histological types. In case B, the presence in both HER2+ and HER2− areas of shared new allelic abnormalities strongly suggests that the tumour derives from a single clone, HER2+ area and HER2− area being two different subclones. Therefore, in case B, HER2 amplification may be a progression event in a clonal tumour cell population, as recently reported (Hanna *et al*, 2007; Kostopoulou *et al*, 2007; Cottu *et al*, 2008; Apple *et al*, 2009).

In conclusion, we demonstrate an excellent concordance between IHC and Q-RT–PCR for HER2 status assessment in breast tumours. Rare discordances may be due sometimes to intra-tumour heterogeneity. The association of these two methods in IHC equivocal cases or even in all tumours may be a reliable and moderate-cost strategy for HER2 status assessment. The quantitative nature of Q-RT–PCR could also provide clinically relevant informations, allowing tailored treatment according to the amplitude of HER2 overexpression in breast cancers.

## Figures and Tables

**Table 1:** Studies comparing Q-RT–PCR and other HER2 assessment methods

**References**	**No. of cases**	**IHC antibody concordance or *κ* with Q-RT–PCR**	**FISH–CISH method concordance with Q-RT–PCR**	
Cronin *et al* (2004)	62	Not specified	Not evaluated	FFPE specimens
		100%		
Gjerdrum *et al* (2004)	30	HercepTest	Vysis PathVysion HER2 kit	Microdissected FFPE specimens
		—a	—a	
Ginestier *et al* (2004)	94	Multiple antibodies (A0485, TAB250, CB11, 3B5 and HercepTest)	ERBB2 FISH Pharm DX kit and Vysis PathVysion HER2 kit	FFPE specimens
		82–93% depending of the Ab	84–88%	
Vanden Bempt *et al* (2005)	32	Multiple IHC procedures	Multiple FISH procedures	Frozen and FFPE specimens
		100%	96.8%	
Vinatzer *et al* (2005)	43	HercepTest	Vysis PathVysion HER2 kit	Frozen specimens
		86.4%	76.6%	
Esteva *et al* (2005)	149	neu Ab-8; *κ*=0.6±0.08	Not evaluated	Frozen specimens
Bossard *et al* (2005)	44	A0485 and CB11	Not evaluated	Frozen specimens
		84%		
Kostopoulou *et al* (2007)	49	CB11	Vysis PathVysion HER2 kit	Frozen specimens
		—a	93.8%	
Barberis *et al* (2008)	44+55	HercepTest	Vysis PathVysion HER2 kit	Frozen and FFPE specimens
		82%	72.7%	
Cuadros *et al* (2010)	80	HercepTest	ERBB2 FISH Pharm DX kit	Frozen specimens
		—a	—a	

Abbreviations: CISH=chromogenic *in situ* hybridisation; FFPE=formalin-fixed paraffin embedded; FISH=fluorescent *in situ* hybridisation; IHC=immunohistochemistry.

a These studies used both Q-RT–PCR and one other method, but did not precisely evaluate concordance/discordance between techniques.

Note that most studies considered score 2+ as positive for the calculations, whereas 2+ cases are very poorly amplified.
